# Eukaryotic pathways targeted by the type III secretion system effector protein, BipC, involved in the intracellular lifecycle of *Burkholderia pseudomallei*

**DOI:** 10.1038/srep33528

**Published:** 2016-09-16

**Authors:** Wen-Tyng Kang, Kumutha Malar Vellasamy, Jamuna Vadivelu

**Affiliations:** 1 Department of Medical Microbiology, Faculty of Medicine, University of Malaya, 50603, Kuala Lumpur, Malaysia

## Abstract

*Burkholderia pseudomallei*, the etiological agent for melioidosis, is known to secrete a type III secretion system (TTSS) protein into the host’s internal milieu. One of the TTSS effector protein, BipC, has been shown to play an important role in the *B. pseudomallei* pathogenesis. To identify the host response profile that was directly or indirectly regulated by this protein, genome-wide transcriptome approach was used to examine the gene expression profiles of infected mice. The transcriptome analysis of the liver and spleen revealed that a total of approximately 1,000 genes were transcriptionally affected by BipC. Genes involved in bacterial invasion, regulation of actin cytoskeleton, and MAPK signalling pathway were over-expressed and may be specifically regulated by BipC *in vivo*. These results suggest that BipC mainly targets pathways related to the cellular processes which could modulate the cellular trafficking processes. The host transcriptional response exhibited remarkable differences with and without the presence of the BipC protein. Overall, the detailed picture of this study provides new insights that BipC may have evolved to efficiently manipulate host-cell pathways which is crucial in the intracellular lifecycle of *B. pseudomallei*.

*Burkholderia pseudomallei* is an aerobic, Gram negative soil-dwelling bacillus which is the causative agent of melioidosis, a life-threatening disease in humans^1^. Melioidosis presents an array of clinical symptoms ranging from acute to chronic localised infection which impose difficulties for both the bacteriologist and clinicians in differential diagnosis for other common infectious diseases^2^. This disease has been classified as an emerging disease with the steady increase in the number of patients over the past few years. Complete eradication of *B. pseudomallei* from melioidosis patients is difficult with a high recurrence rate. The infection of a host often depends on the ability of this pathogen to inject the virulence factors directly into the cytosol of the host cell with the aid of the type three secretion systems (TTSS)^3^.

TTSS has been reported to play a crucial role in the pathogenicity of many Gram-negative bacteria. The cocktail of proteins which are injected into the eukaryotic host cells are known as the type three secretion effectors^4^. The TTS effectors are of special interest in studies of host-pathogen interaction because these proteins could subvert the host cell metabolic systems using mechanisms such as molecular mimicry or maybe functionally redundant. According to Sun and Gan^5^, the function of TTS effectors in *B. pseudomallei* has not been completely defined except for two of the effectors, BopA and BopE. In this study, our interest is mainly on one of the TTS effectors, *Burkholderia* invasion protein C (BipC), which is also the homologue of *Salmonella* SipC and *Shigella* IpaC^6^. Protein bioinformatics predicted that BipC is one of the hydrophobic translocon and may interact with BipB to form the membrane pore in order to inject effector proteins in the host cell^7^. However, it is conceivable that this protein has the effector functions that interfere with the host cell machinery^5^. In a previous study by our group, it was shown that BipC was present in the secretome of *B. pseudomallei* laboratory culture and was found to be immunogenic as confirmed by reactivity to mice anti-*B. pseudomallei* sera^8^. In addition, we demonstrated that BipC is important in bacterial adherence, invasion, and intracellular survival in epithelial cells *in vitro,* and the *bipC* mutant also demonstrated dramatically attenuated virulence in a murine model of melioidosis^9^. Despite that, very little is known about the mechanisms that underlie the virulence of *B. pseudomallei*.

In the recent years, a number of gene expression analyses have been performed using the microarray platform to gain insight into the host response to *B. pseudomallei* infection^10^,^11^. These transcriptional analyses may be useful to further understand the mechanisms of host defense and the strategies employed by this pathogen to circumvent the host protection system^12^. The differential expression profile of the host in the presence or absence of the effector protein will be used to identify the regulatory mechanisms and pathways related to bacterial pathogenesis. To date, a complete picture of the differences between the host responses to this pathogen, with and without the expression of the TTS effector is still not available. Hence, this study provides an opportunity to develop an in-depth comprehensive understanding of the host transcriptional responses to *B. pseudomallei* BipC. We hypothesise that the interaction of BipC with the targeted pathways in eukaryotic cells may either directly or indirectly be related to the virulence of the pathogen in the infected hosts. To address this, an acute melioidosis infection mouse model was developed and transcriptional analysis in the organs isolated from mice infected with pathogenic *B. pseudomallei* K96243 wild type strain (with BipC expression) and BM16 (without BipC expression) was performed in this study.

## Results

### Phenotypic microarray of *B. pseudomallei* WT and depleted BipC strains

Phenotypic microarray (PM) analysis allowed high-throughput screening of the phenotypic differences between the WT and *bipC* mutant under a wide range of conditions. Comparison between the WT and BM16 strains, demonstrated noticeable differences in the metabolic profiles of both strains (Tables 1 and 2). However, the carbon catabolism capacity, determined from the heat map, demonstrated no significant difference between the WT and BM16 strains tested, whereby 31% (59) of 190 carbon substrates tested were utilised (see Supplementary Fig. S1; corresponding numerical data are shown in Supplementary Table S1). On the other hand, the nitrogen metabolism capacity was low for both WT and BM16, recording the utilisation of only 12% (11) of the 95 nitrogen substrates tested. The metabolism of phosphorus and sulphur of both WT and BM16 strains were extremely low (0.01%) and no significant difference was observed for all the 95 substrates tested. The primary difference observed in the PM analysis was decreased metabolism in the BM16 on most of the nitrogen sources tested. It is notable that all the depleted nitrogen sources involved only the amino acids. Despite its poor growth in most of the nitrogen sources, it was rather intriguing that *bipC* showed comparable growth with the WT strain in some of the carbon sources tested.

### Infection murine model of melioidosis

In this study, BALB/c mice were infected with *B. pseudomallei* K96243 wild type (WT) and *bipC* mutant (BM16) strains via the intraperitoneal (i.p.) route. Based on the LD_50_ (Supplementary Fig. S2), the time to achieve 100% death from 10^4^–10^6^ CFU of WT was four days, two days, and one day, respectively, while 60% of mice injected with 10^3^ CFU survived until the end of the observation period. It was found that upon infection, the mice infected with a dosage of >10^4^ CFU of WT displayed symptoms of disease such as lethargy and had ruffled fur before capitulating to infection. Infection dose of 10^4^ CFU was chosen for the subsequent experiments as it was shown that the infection with 10^5^ and 10^6^ CFU of WT were too virulent.

**Figure 1:**
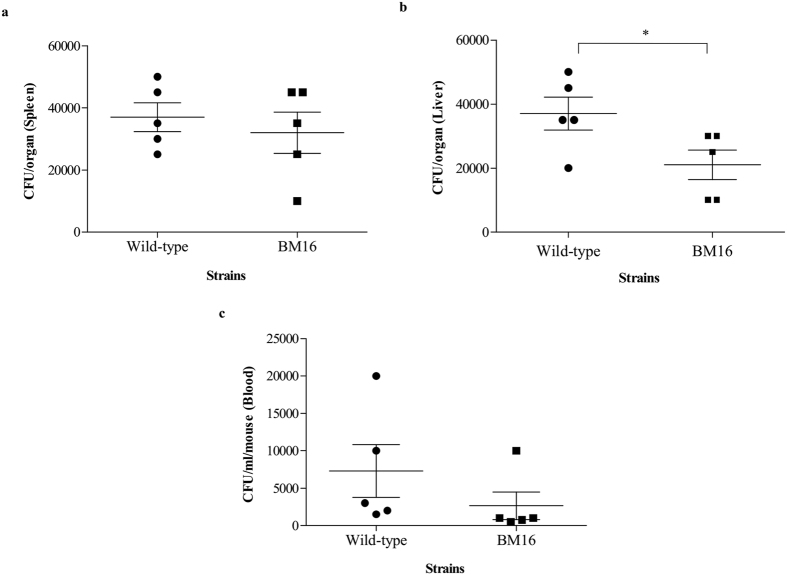
Bacterial burdens in the organs and blood of BALB/c mice. The bacterial load in the (**a**) spleen, (**b**) liver, and (**c**) blood of BALB/c mice at 24 hours after intraperitoneal inoculation with approximately 10^4 ^CFU of *B. pseudomallei* K96243 (wild type) and *bipC* mutant strains (BM16). There were no colonies found in any of the organs or blood samples from the control mice. Each point on the graph indicates the CFU measured in a single mouse (n = 5). The horizontal marks show the average CFU ± SEM. *p < 0.05 using Student’s t-test.

The bacterial loads in various organs and blood in BALB/c mice infected with *B. pseudomallei* WT and BM16 were determined in order to characterise the murine model of melioidosis (Fig. 1). At 24 hours post-infection (hpi), the average BM16 load in the spleen (3.5 × 10^4 ^CFU/organ) was higher than the liver (2.0 × 10^4 ^CFU/organ), while the WT load in both organs showed similar average of 4.0 × 10^4 ^CFU/organ (Fig. 1(a,b)). Statistical analysis revealed that no significant differences were observed in the bacterial load in the spleen, whereas there were significant differences observed in the liver (*p* = 0.048). Viable *B. pseudomallei* were also detected in the blood of the infected mice at lower numbers (10^3 ^CFU/ml) (Fig. 1(c)). Taken together, the presence of *B. pseudomallei* in various organs and the blood confirmed that the experimental melioidosis model in BALB/c mice was successfully developed for this study.

### Host transcriptional responses profiles obtained from *B. pseudomallei* WT and BM16 mutant infection

**Figure 2:**
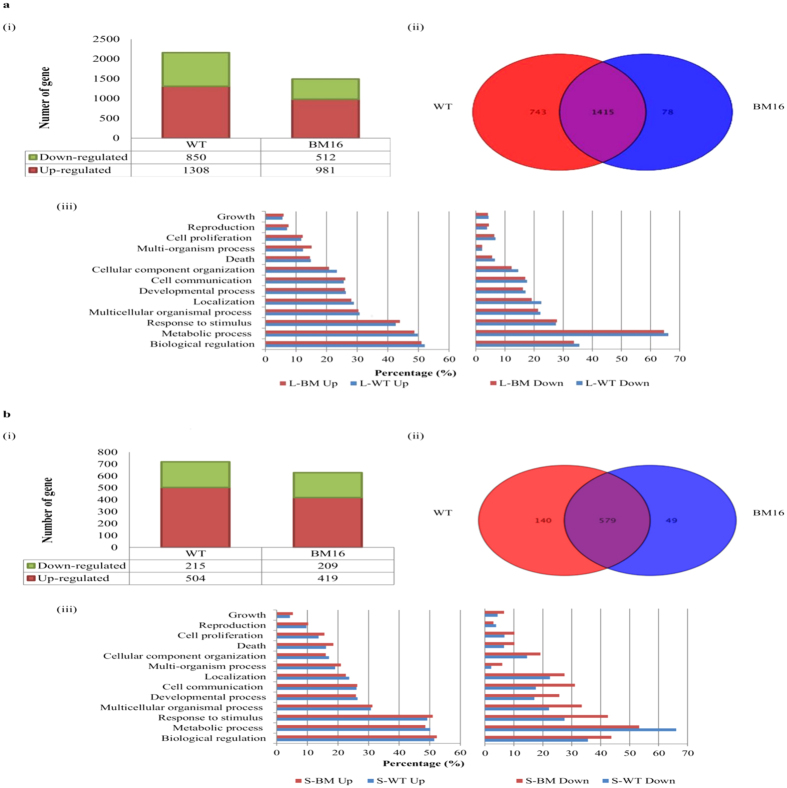
Number and functional annotation of the mouse gene expression at 24 hpi with *B. pseudomallei* wild type and *bipC* mutant infection. (i) Total number of significant differently expressed genes (*p* < 0.01) following infection with *B. pseudomallei* WT and BM16, (ii) Venn diagrams displaying the number of differentially expressed genes in (**a**) liver (L) and (**b**) spleen (S), respectively, and (iii) gene onthology (GO) categories for both WT and BM16 up- and down-regulated genes.

In order to compare the gene expression profiles of *B. pseudomallei* WT and BM16, mouse whole-genome microarray from Agilent was used to investigate the global changes of gene expression in the infected organs. The transcriptome of infected liver and spleen over an infection period of 24 hours were compared with the uninfected tissues to identify gene expressions that are altered in response to the WT and BM16 infection (Fig. 2). Statistical analysis (*p* < 0.01) combined with a 2-fold variation cut-off indicated that a total of 2,158 genes and 1,493 genes were differentially expressed in the liver infected with WT and BM16, respectively (Fig. 2a(i)). On the other hands, a total of 719 genes and 628 genes were differentially expressed in the spleen infected with WT and BM16, respectively (Fig. 2b(i)). Noticeably, WT infection in BALB/c mice resulted in a higher number of differentially expressed genes in the liver and spleen as compared to the BM16 strain. Venn diagrams were generated to examine the distribution of differentially expressed genes in different experimental conditions. In the liver, WT regulates 743 genes whilst BM16 regulates 78 genes, all exclusively, whereas in the spleen, only 140 (WT) and 49 (BM16) genes were exclusively regulated (Fig. 2a(ii)). Thus, these results indicated that BipC protein secreted by TTSS3 may be responsible for the induction and repression of transcriptional responses in the host cells.

### Characterisation of differently expressed genes between the *B. pseudomallei* WT and BM16 mutant infection

Gene function enrichment analysis of the differently expressed genes, that were regulated by the infection with the WT and BM16 mutant, could assist in interpreting the dominant functions controlled by those genes in this study. Web-based software GOTerm Finder was used to evaluate the biological processes of the differentially expressed genes. Figure 2a(iii) showed the important Gene Ontologies (GO) with *p* < 0.05 and the analysis revealed that the majority of differentially expressed genes were mainly involved in biological regulation, metabolic process, and response to stimulus. A similar pattern of involvement in biological process was observed among the up-regulated genes under both the WT and BM16 conditions in liver and spleen. Among the down-regulated genes in the liver, similar percentage of genes in the WT and BM16 conditions were found to be involved in all the different biological processes. However, comparison among the down-regulated genes in spleen showed noticeably higher percentages of genes in BM16 condition were involved in almost all the biological processes. For WT in spleen, majority of the down-regulated genes (66.1%) were involved in host cellular metabolism process. These results further addressed the contribution of BipC to the *B. pseudomallei* TTSS3 mediated stimulation of transcriptional response *in vivo*.

### Functional classification of BipC and pathway analysis

The list of all significant differentially expressed genes was analysed for the associated Kyoto Encyclopedia of Genes and Genomes (KEGG) pathways using the GeneTrail and GATHER software. This pathway analysis allowed further investigation of the over- or under-represented functional activities of BipC. Significantly impacted pathways and corresponding *p*-value are presented in Tables 3 and 4. Results of the analyses indicated that infection with the WT and BM16 regulate higher number of pathways in the liver as compared to the spleen. Based on the statistical analysis of KEGG pathways, the number of genes involved in cellular processes such as cell motility, transport and catabolism, and signal transduction were significantly higher in the liver following infection with the WT. Besides, the analysis revealed that several pathways were significantly dysregulated by the loss of BipC involved in cellular processes, including the regulation of actin cytoskeleton and p53 signalling pathway; as well as the bacterial invasion of epithelial cells and MAPK signalling pathway.

**Figure 3:**
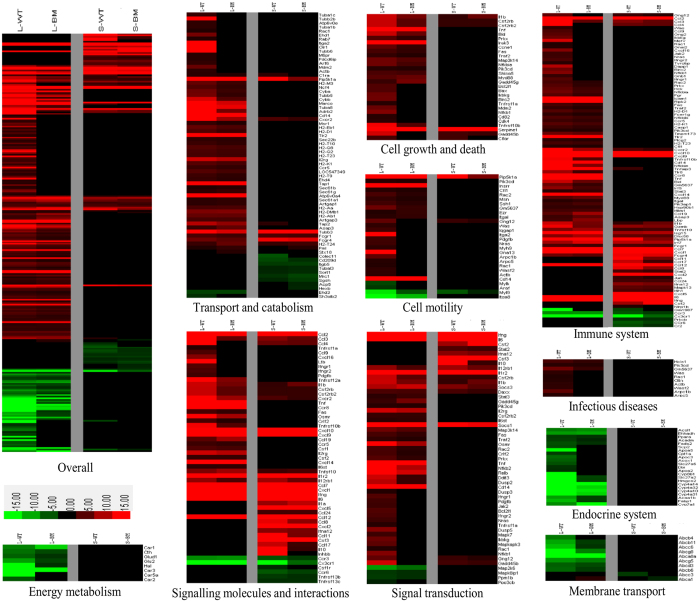
Global gene expression patterns of *B. pseudomallei* wild type and mutant infection. Heat map depicts fold changes of the differentially expressed genes observed in the liver (L) and spleen (S) following infection with *B. pseudomallei* WT and BM16 mutant strains. The key on the bottom left of the panel represents the relative differential expression corresponding to the colours in the heat map. Red indicates up-regulation and green indicates down-regulation.

In addition, the specific genes involved in each of the pathways were categorised according to their functional categories and fold change relative to uninfected mice are presented as a heat map (Fig. 3). The heat map hierarchical clustering analysis in this study revealed different patterns of gene regulation for liver and spleen. Infection with both the WT and BM16 in BALB/c mice showed a higher number of differentially expressed genes in the liver (229 genes) compared to the spleen (75 genes). Notably, most of these differentially expressed genes in the liver were found to be up-regulated (177 genes). Interestingly, several cell motility-related genes, such as actin (Actb), actin related protein 2/3 complex (Arpc5 and Arpc1b), guanine nucleotide binding protein (Gna13), integrin (Itga2), neuroblastoma ras oncogene (Nras), platelet derived growth factor (Pdgfb), RAS-related C3 botulinum toxin (Rac1), GTPase activating protein (Iqgap1), myosin (Myh9) and WAS protein family (Was and Wasf2) were significantly up-regulated in the infection with the WT but absent in the infection with BM16 mutant. Moreover, the up-regulated genes TNF receptor superfamily member (Fas) and tumour necrosis factor (Tnf) were found to be highly expressed in the liver of mice infected with the WT as compared to the BM16 (Supplementary Table S2). Both of these genes were known as transcription regulators and found to co-target many of the pathways which are implicated as regulators for the signalling molecules and interaction (cytokine-cytokine receptor interaction), signal transduction (MAPK signalling pathway), cell growth and death (apoptosis), and immune system (natural killer cell mediated cytotoxicity).

### IPA software for network analysis of BipC

**Figure 4:**
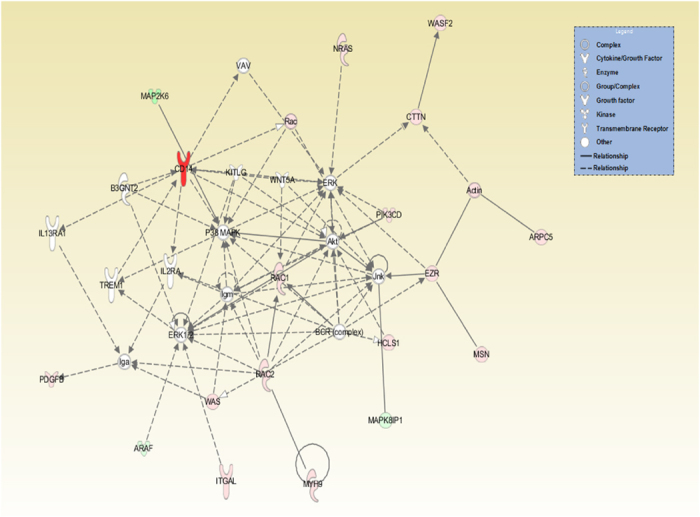
Ingenuity pathway analysis network for genes comparing the liver from mice infected with *B. pseudomallei* wild type to *bipC* mutant. This network illustrated the relationships among significant differentially expressed genes of regulation of actin cytoskeleton. Functional classes of the gene products are presented using various shapes. Intensity of the red and green colour reflects the degree of up- or down-regulation, respectively.

**Figure 5:**
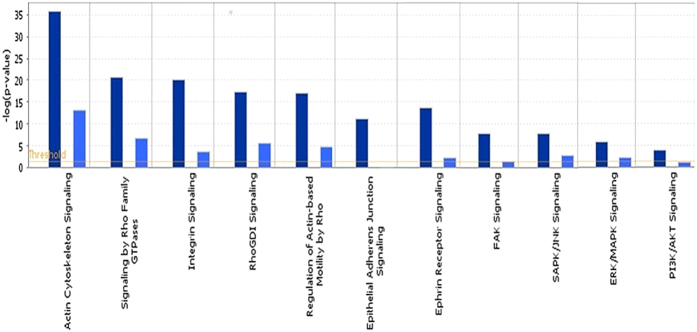
Canonical pathway identified by IPA associated with responsive genes in liver infected with *B. pseudomallei* wild type and mutant. The pathway involved in the cellular processes for the differentially expressed genes between WT and BM16. Dark blue representing WT and light blue representing BM16.

IPA software used to further investigate the functional activities of BipC through the networks of biologically related genes that are co-regulated or differentially regulated in response to WT and BM16 infection demonstrated the relevance of cell motility-related genes which were identified in the WT liver (Fig. 4). The network presented p38 mitogen-activated protein kinases (MAPK) pathway, c-Jun N-terminal kinase (JNK) signal transduction pathway, extracellular signal-regulated kinase (ERK) pathway, and serine/threonine kinase (AKT) signalling pathway in central positions. Canonical pathways determined that the pathways were significantly different between WT and BM16 (Fig. 5). These pathways mainly involved in the cellular movement, haematological system development and function, and immune cell trafficking which consistent with the finding of GenTrial.

### Validation of microarray findings with real-time PCR

**Figure 6:**
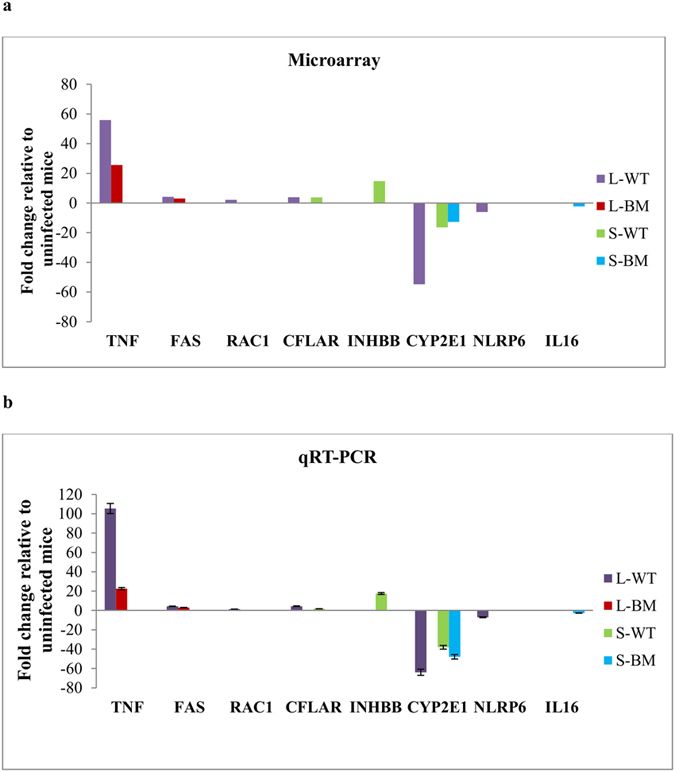
Real-time quantitative PCR validation of microarray data. Regulation of genes used for validation in (**a**) microarray and (**b**) qRT-PCR assays in the liver (L) and spleen (S) following infection with *B. pseudomallei* WT and BM16 mutant strains. The data represent the mean ± standard deviation from three biological replicates. TNF, Tumor necrosis factor; FAS, TNF receptor superfamily member 6; RAC1, RAS-related C3 botulinum substrate 1; CFLAR, CASP8 and FADD-like apoptosis regulator; INHBB, Inhibin beta-B; CYP2E1, Cytochrome P450, family 2, subfamily e, polypeptide 1; NLRP6, NLR family, pyrin domain containing 6; IL16, Interleukin 16.

Validation of the microarray results were performed using quantitative real-time polymerase chain reaction (qRT-PCR). To verify the microarray results, eight target genes from different functional categories that were differentially expressed were selected and a housekeeping gene glyceraldehyde 3-phosphate dehydrogenase (*GAPDH*), was used for normalisation (Fig. 6). Samples previously used for the microarray experiments were taken for the qRT-PCR analyses. The genes analysed were confirmed as up- or down-regulated as an independent measure of differential gene expression through qRT-PCR (Fig. 6(b)). All the genes used in validation had the same pattern of regulation as that of the microarray results (Fig. 6(a)). Thus, this result verified that the reliability of the designed microarray for high throughput screening of the transcriptome of mouse in this study.

## Discussion

Herein, we described the first phenotypic microarray and genome-wide study of TTS effector regulated gene expression in *B. pseudomallei*. The high-throughput PM technology was used for simultaneous comparison of a range of substrate utilisation using the respiration rate as an indication of growth^13^. Analysis of the PM data found that the WT and BM16 showed phenotypic changes based on their ability to utilise a wide range of nutrient sources, especially the carbon and nitrogen sources. Interestingly, the *bipC* mutant was shown to have pleiotropic phenotypes on nitrogen sources, which may play a role as transporters of amino acids. Many microorganisms utilise amino acids as a source of energy or nitrogen for the biosynthetic purposes. A study by Yabu^14^,^15^,^16^ has determined that D-amino acids are rapidly taken up by mycobacteria while the L-forms are transported at a much lower rate. These results can be attributed to the specificity of the inner-membrane transporters for the natural form of amino acids. Competition between the pathogen and macrophages for arginine has been suggested to contribute to the outcome of infection^17^. Moreover, *Salmonella* was shown to modulate cellular trafficking processes in a SPI2 T3SS-dependent manner by gaining access to a supply of nutrients and membrane materials for the formation of a tubular network of membrane structures called SIFs that connect to the small colony variants in the infected cell^18^. Hence, the reduced rate of respiration due to some of the amino acids as nitrogen sources may indicate that BipC is required for the transport of amino acids in the cells.

In addition, gene expression of mice infected with *B. pseudomallei* WT and *bipC* mutant was profiled as it may reveal the mechanisms by which the host responds to *B. pseudomallei* and to aid further understanding of the role of BipC in virulence of *B. pseudomallei.* Prior to the gene expression study, a murine melioidosis model was established, using the intraperitoneal route of infection, in order to enhance our knowledge on the manifestation of this disease in the early stages of infection. As the bacteria are able to survive in macrophages, it is possible that the bacteria may not only remain in one organ but may also be present in other organs of the mouse^19^. As such, in this study, both the liver and spleen, which typically have the highest bacterial loads during the experimental melioidosis^20^, were further selected for the transcriptomic study. The differential gene expression in *B. pseudomallei*-infected liver relative to the control was found to be more intense than that in the infected spleen. As the liver is responsible for the protection of the host from infectious diseases, thus, it is reasonable to expect that the expression of particular genes under this stressful condition would be altered^21^.

In our study, more than 1,000 mouse genes demonstrated significant differential regulation at 24 hpi, indicating the ability of BipC to alter the responses of the host towards *B. pseudomallei* infection. This definitely suggests the importance of BipC in *B. pseudomallei* infection. *B. pseudomallei* WT infected host was shown to overexpress many signal transduction genes as compared to the BM16 infection. Genes involved in signal transduction pathways were up-regulated in both the liver and spleen of the mice. In the liver infected with WT, MAPK signalling pathway was shown to be significantly up-regulated with the up-regulation of genes such as, dual specificity phosphatase (Dusp 3 and 5), mitogen-activated protein kinase (Mapkapk3 and Mapk7), tumour necrosis factor receptor superfamily (Tnfrsf1a), inhibitor of kappaB kinase gamma (Ikbkg), nuclear factor of kappa light polypeptide gene enhancer in B cells (Nfkb1), neuroblastoma ras oncogene (Nras), guanine nucleotide binding protein (Gng12), platelet derived growth factor (Pdgfb), ras homolog gene family (Rhoc), RAS-related C3 botulinum (Rac1), and growth arrest and DNA-damage inducer (Gadd45b). Saadat *et al.*^22^ have reported that *H. pylori* CagA specifically interacts with PAR1/MARK kinase for disorganisation of gastric epithelial architecture resulting in mucosol damage, inflammation, and carcinogenesis. By analogy, we believe that BipC could activate the MAPK kinase, which has an essential role in the epithelial cell polarity. Besides that, signalling molecules are preferentially targeted by most of the pathogens due to the presence of their ability to globally regulate many cellular processes^23^. Thus, the outcome of *B. pseudomallei* infection is determined by the highly synchronised interaction of its virulence factors with the signal transduction pathways in host cells ultimately leading to diverse cellular response^24^.

Wessler^25^ demonstrated that activation of the complex network of signal transduction pathways, including adaptor protein, host cell kinase, Rac and Rho family of small GTPases, and actin binding proteins lead to the regulation of the actin cytoskeleton, which is involved in the invasive growth, as well as proliferation processes in the host cytoplasm^26^,^27^,^28^. In comparison between the infections of the WT versus BM16, Rho-family GTPases (Rac1 and Rhoc) were shown to be activated in the liver infected with WT but not in the BM16 infection. The Rho-family GTPases participate in regulation of the several cellular processes, including regulation of the actin cytoskeleton, assembly of intercellular junctions, cell adhesion, and membrane trafficking^29^,^30^. In mammalian cells, specific activation of Rho, Rac, and Cdc42 leads to characteristic rearrangements of the actin cytoskeleton^31^. Usually, activation of Cdc42 and Rac1 are associated with the formation of filopodia and lamellipodia, respectively^32^,^33^. As activation of these proteins lead to specific changes in key cellular functions (*i.e.* regulation of actin cytoskeletal), BipC may possess the capability in activating RhoGTPase signalling cascades leading to cytoskeletal rearrangements and enable *B. pseudomallei* to precisely manipulate host cell physiology. According to Raftopoulou and Hall^34^, the GTPases play a crucial role in several cellular processes, including cell migration, cytokinesis, phagocytosis, and cell-matrix contacts. Therefore, the capacity to activate Rac1 and RhoGTPase (Rhoc) via BipC is particularly important for *B. pseudomallei* pathogenesis.

In host-pathogen interactions, the critical initial step is the bacterial adherence and invasion of the host cells. The actin cytoskeleton and its regulatory system are often exploited by pathogens to adhere to and invade the host cells^35^,^36^. Based on our gene expression data, most of the up-regulated genes which are associated with the various cellular processes including adhesion and invasion of host cells were absent in the liver and spleen following BM16 infection. Actin cytoskeleton plays a major role in many aspects of the biological processes, such as cell-cell interactions, cell motility, formation of immunological synapse, endocytosis, phagocytosis, and intracellular protein trafficking^37^,^38^,^39^. Therefore, it is noteworthy that the regulation of the actin cytoskeleton by BipC is important for the host-pathogen interactions. This observation is in line with our previous study whereby *B. pseudomallei* BipC was shown to induce actin-tail formation and is involved in the disruption of intracellular adhesions, invasion, intracellular survival, and the escape from the phagosome^9^.

The subversion of the host actin cytoskeleton components can trigger an immune response against the pathogen^40^. Differential expressions of immune responses associated genes were highly evident from the 24 hpi with the WT and BM16. The presence of BipC in the WT strain marked up-regulation of several pro-inflammatory response genes in our study. The apoptosis inhibitor genes including tumour necrosis factor receptor (Tnfaip3) and CASP8/FADD-Like apoptosis regulator (Cflar) appear to be significantly up-regulated in both liver and spleen infected with WT but not at all in the organs infected with BM16. The Cflar is one of the crucial apoptotic inhibitor associated with apoptosis and cell survival. Remarkably, this protein has the ability to block apoptosis induced by large amounts of extracellular TNF family^41^. Study by Faherty *et al.*^42^ has determined that *Shigella* has the ability to induce apoptosis via a classical extrinsic TNF-α receptor-mediated caspase-8 pathway which involves the Tnfaip3 and Cflar. Interestingly, both of these genes were also induced in our microarray analysis. The suppression of early apoptosis in the host cell may be beneficial for the virulent microbe. In addition, the increased anti-apoptotic responses to the virulence strain infection correlate with the induction of higher transcription of nuclear factor of kappa light polypeptide gene (Nfkb1), a gene which may have anti-apoptotic effects^43^,^44^. This Nfkb1 had also significant induction in the WT infected cells. TNF-α-dependent apoptosis during WT infection may be countered by Cflar-and Nfkb-dependent anti-apoptotic responses. Based on the array results, BipC may be required for the induction of apoptosis which allows the pathogen to survive in the host.

The subfamilies of inflammatory mediator (Casp1, Casp4) were up-regulated in response to infection with both *B. pseudomallei* WT and BM16. *B. pseudomallei* virulence factors such as TTSS factors have been shown to be involved in triggering the inflammasome-dependent caspase-1 activation^45^. A number of Gram-negative bacteria such as *Listeria spp., Pseudomonas spp.,* and *Salmonella spp.* were found to have the ability to induce caspase-1 activation and execute macrophage cell death via amplification of inflammatory responses^46^. The activation of caspase cascades and stimulation of inflammation by effector proteins are important in controlling the proliferation of intracellular pathogens in the host cell^47^,^48^. Moreover, many genes associated with the cell death were up-regulated in the WT infection as compared to BM16 infections. Elevation of this cell death associated genes such as tumour necrosis factor receptor (Tnf), Fas associated factor (Fas), BH3 interacting domain death agonist (Bid), myeloid differentiation primary response gene (Myd88), interleukin gene (Il1b), colony stimulating factor (Csf2rb), and nuclear factor of kappa light polypeptide gene (Nfkb1), leads to the up-regulation of the apoptosis signalling. The caspase-1 dependent macrophage death induced by *B. pseudomallei* has previously been reported by Sun *et al.*^49^. The induction of interleukin gene (Il1b) was also observed in this study. In addition, p53 signalling pathway was up-regulated with the up-regulation of cyclin (Ccne1), growth arrest and DNA-damage factor (Gadd45g), serine peptidase inhibitor (Serpine1), and Shisa5. However, the expression of genes in BM16 infection was shown to be lower as compared to the WT. Many genes associated with the cell death such as tumour necrosis factor receptor (Tnfrsf1a), inhibitor of kappaB kinase (Ikbkg), kinases (Cd82 and Cdk2), and apoptosis regulator (Bcl2l1 and Birc2) were only up-regulated in WT infection and showed no expression in BM16 infection. Both *Shigella flexneri* and *Salmonella Typhimurium* uses its TTSS to inject the effectors, which binds to caspase-1, and activates its proteolytic function, thereby inducing apoptosis^46^. Thus, consistent with our observation, *B. pseudomallei* is also postulated to inject BipC and cooperate with caspase-1 to induce apoptosis.

Intracellular pathogenic bacteria share the ability to evade host immunity by impairing trafficking of endocytic cargo to lysosomes for degradation^50^. Both Rab and ARF families are crucial for membrane trafficking^51^. Recently, *Chlamydia trachomatis* Inc protein was identified as a regulator of species-specific Rab GTPase and induced vesicular trafficking inside the eukaryotic host cell^52^. Besides, vesicular trafficking via ADP-ribosylation factor 1 (Arf1) was found to be mainly required for inclusion membrane growth and stability. In this study, we also found that the expression of genes associated with the member RAS oncogene family (Rab7 and 35) and ADP-ribosylation factor (Arf3 and 6) in the liver infected with WT. Costa and colleagues^53^ showed that the *Salmonella enterica* secreted effector SopD2 mediates trafficking suppression by binding the host regulatory GTPaseRab7 and inhibiting its nucleotide exchange. Besides that, *Salmonella spp.* effector proteins, SopB also promotes invasion by manipulating eukaryotic vesicular trafficking probably to induce fusion of intracellular vesicles to the cell membrane at the entry site^54^. Thus, it seems that BipC may have the ability to interfere with intracellular trafficking which is essential for *B. pseudomallei* pathogenesis.

The regulation of actin cytoskeleton, MAPK signalling pathway, p53 signalling pathway, and bacterial invasion of epithelial cells were significant at the stage of WT infection compared to BM16 infection in our study. We found that these pathways were closely related to cellular processes, signal transduction, and are involved in the cellular trafficking. These findings provide not only key insight into the mode of immune evasion by this pathogen but also functional information of BipC which influences eukaryotic cell pathways. Overall, the eukaryotic signalling pathways targeted by BipC were determined either directly or indirectly in modulating the intracellular behaviour of the pathogen which finally leads to the pathogenesis of *B. pseudomallei* infection.

## Methods

### Bacterial strains and growth conditions

*B. pseudomallei* K96243 wild type (WT) and *bipC* mutant strain (BM16) were used in this study. *B. pseudomallei* BM16 mutant was previously constructed through insertional mutagenesis^9^. *B. pseudomallei* WT were grown in Luria-Bertani (LB) broth at 37 °C with 180 rpm agitation for overnight. The mutant strain was grown in LB containing chloramphenicol. The overnight cultures were diluted to 1:100 in LB broth and grown to mid-logarithmic phase until OD_600nm_ of 0.5 (~3 × 10^8 ^CFU per ml) was reached. Ten millilitres of bacterial culture was then centrifuged at 8,000 rpm for 15 minutes and the pellet was resuspended in 10 ml of PBS. The suspension was immediately diluted to the desired bacterial titre (10^4 ^CFU per ml) in sterile PBS.

### Phenotypic microarray (PM)

The phenotypic analyses were performed using BioLog phenotypic microarray microplate PM1–2 (carbon sources), PM3 (nitrogen sources), and PM4 (phosphorus and sulphur sources), according to the manufacturer’s instruction (Biolog, Hayward, California, USA). In brief, *B. pseudomallei* WT and *bipC* mutant strains were cultured from glycerol stocks and passaged three times before the phenotypic microarray experiment. A single colony of these bacterial was picked and inoculated into the Biolog inoculating fluid (IF-0). Hundred microliters of the cells were transferred into each well and incubated in an OmniLog instrument (Biolog, Inc., Hayward, California, USA) at 37 °C for 48 hours. The substrate utilisation was measured via the reduction of a tetrazolium dye forming a purple formazan and is indicative of active cellular respiration, while the negative control wells remained colourless^13^. The cellular respiration of wild type and mutant were recorded and the data were analysed using the OmniLog software. The option of A1 zero was selected during data processing to deduct the background signal with reference to the A1 negative control well in each plate. Three biological replicates were conducted for each strain.

### Animal model

Female, 6- to 7-week-old, pathogen-free healthy BALB/c mice were purchased from Monash University, Malaysia. They were housed in polysufone cages with a bedding of paper shavings under a strict 12 hours light/dark cycle. Mice were fed a diet of commercial pellets and distilled water, and maintained under specific-pathogen-free conditions. All animal experiment procedures were performed in accordance with the University Malaya animal ethics guidelines for the humane use of laboratory animals. The procedures in this animal study were approved by the University Malaya Institutional Animal Care and Use Committee (IACUC) (Animal ethics protocol PAT/05/11/2007/WKT[R]). All the mice were monitored daily by our qualified personnel throughout the experiments and humanely euthanised by inhalation of an overdose of isoflurane according to protocols supplied by the University Malaya IACUC to minimise suffering.

### Determination of 50% lethal dose (LD_50_)

Determination of LD_50_ was performed according to Welkos *et al.*^55^ with minor modifications. The median lethal doses (MLD) were determined by the methods of Reed and Muench^56^. BALB/c mice were divided into four groups of five per group and each group was inoculated with the *B. pseudomallei* K96243 strain at different doses (10^3^ to 10^6^ colony forming unit, CFU) via the i.p. route. A group of five mice were inoculated with the bacteria diluted in 200 μl of sterile phosphate-buffered saline (PBS), while the negative control group of five mice were injected with sterile PBS only. The mice were monitored daily (twice a day) for a sign of infection and death throughout the test duration of 14 days. Mice were euthanised according to predetermined humane end point protocols supplied by the University Malaya IACUC at the end of the test duration.

### Mice infection and enumeration of bacteria CFU in organs and blood

The mice infection experiments were performed as previously described with slight modification^57^. For intraperitoneal infection of mice, a group of five mice were inoculated with the appropriated dose of *B. pseudomallei* WT and BM16 suspension in a total volume of 200 μl sterile PBS (1 × 10^4^ CFU/ml), respectively. The control group of five mice were injected with sterile PBS only. Following 24 hours post-infection (hpi), the infected mice blood (500 μl) was collected via cardiac pump using syringe rinsed with EDTA. The blood samples (100 μl) were then plated on Ashdown agar and resulting colonies counted after two days of incubation at 37 °C. Following blood collection, the mice were euthanised by overdose of isoflurane inhalation according to IACUC standard protocol in order to harvest the organs for the determination of bacterial CFU and total RNA extraction. Both liver and spleen were obtained aseptically and homogenised using a tissue homogeniser (T10 Basic Ultra-Turrax, IKA, Staufen, Germany). Homogenised tissue samples were serially diluted and 100 μl of each dilution was plated on Ashdown agar. The bacterial burden was enumerated as CFU per organ.

Total RNA from each organ sample was then rapidly extracted using RNeasy Protect Mini kits (Qiagen, Venlo, Netherlands) following to the manufacturers’ instructions. The isolated RNA samples were then subjected to on-column DNase (Qiagen, Venlo, Netherlands) treatment according to the manufacturer’s protocol. Quantity and quality of the RNA were determined using Nanodrop ND-1000 UV-VS spectrophotometer (Thermo scientific, Wilmington, Delaware, USA) and RNA 6000 Nano LabChip on an Agilent 2100 Bioanalyser (Agilent Technologies, Santa Clara, California, USA).

### Gene expression processing

Microarray experiments were performed using the SurePrint G3 Mouse GE 8 × 60 K BeadChips (Agilent Technologies, Santa Clara, California, USA), containing over 60,000 probes, according to the manufacturer’s instructions. Biotinylated single-stranded cDNA was prepared from a total of 25 ng RNA extracted from each mouse organ (liver and spleen). In brief, mouse cDNA was hybridised to the Mouse Gene Expression array after which the array was washed and scanned with the SureScan microarray scanner (Agilent Technologies, Santa Clara, California, USA) following the manufacturer’s protocols. The fluorescence values for each feature on the array were measured and the resulting image of decoded gene expression data was then extracted using the Agilent Feature Extraction software (Agilent Technologies, Santa Clara, California, USA) for further data analysis. Three biological replicates were performed for each sample.

### Microarray data analysis

Analysis of microarray data was performed using Agilent Feature Extraction (FE) software. This program was used for quality control and to generate signal intensity values from the scans. Following the quality control, raw microarray data were extracted whereby the signal intensity image decoded using BeadArray Reader was converted to numerical data. The raw data was then recovered and subjected to standard normalisation procedure for one-colour array data using GeneSpring GX version 13.0 (Agilent Technologies, Santa Clara, California, USA). Box Plot was then used to check for the presence of any outliers. The normalised data were grouped based on the different experimental conditions (strains and organs) and statistical analysis (One-Way ANOVA) was used to obtain the significant (*p* < 0.05) number of genes that were differentially expressed. Furthermore, the genes were filtered on a Volcano Plot to identify the differentially expressed genes between different experimental conditions with the uninfected mice for both liver and spleen with the cut-off of two fold change and *p*-value (<0.01). Venn diagram was then generated to compare the number of genes significantly changed in each set of experimental conditions.

### Gene ontology enrichment and pathway analysis

The set of differentially expressed genes was analysed using various free web-based software that are available. The GOTerm Finder (http://go.princeton.edu/cgi-bin/GOTermFinder), GATHER (http://gather.genome.duke.edu/), GeneTrail (http://genetrail.bioinf.uni-sb.de/), and Ingenuity Systems (IPA) (http://www.ingenuity.com) were used to determine the Gene Ontology (GO) and Kyoto Encyclopedia of Genes and Genomes (KEGG) from a large amount of the microarray data. The Cluster 3.0 and Java Treeview V1.1.3 softwares were used for hierarchical clustering and visualisation of the differentially expressed genes, respectively.

### Quantitative Real-Time PCR (qRT-PCR) analysis

RNA samples used for microarray study were subjected to real-time PCR analysis for validation of the microarray result. Nine genes, including eight genes that were significantly regulated in the microarray analysis and a reference gene, were identified to be used for the validation. The primer sets used in this study are listed in Supplementary Table S3. Real-time quantitative PCR was performed on 10 ng cDNA in a final volume of 20 μl using QuantiNova SYBR green PCR kit (Qiagen, Venlo, Netherlands) according to the manufacturer’s instruction and primers at a final concentration of 1 μM. The thermal steps of qRT-PCR used are of the manufacturer’s recommendations. Normalisation was performed against housekeeping gene *GAPDH* in order to control for variation in RNA concentration. The raw Ct values were obtained using Bio-Rad CFX manager (BioRad Laboratories, Hercules, California, USA). The fold changes of the expression of the genes under both the WT and mutant conditions were calculated using standard delta-delta-Ct method^58^. Quantitative experiment was performed in triplicate according to the manufacturer’s guidelines.

## Additional Information

**How to cite this article**: Kang, W.-T. *et al.* Eukaryotic pathways targeted by the type III secretion system effector protein, BipC, involved in the intracellular lifecycle of *Burkholderia pseudomallei. Sci. Rep.* **6**, 33528; doi: 10.1038/srep33528 (2016).

## Supplementary Material

Supplementary Information

## Figures and Tables

**Table 1:** PM conditions where WT respired more than BM16.

Plate	Well	Item tested	Mode of Action
PM01	B01	D-Serine	C-Source, amino acid
PM03B	A12	L-Glutamic Acid	N-Source, amino acid
PM03B	B01	L-Glutamine	N-Source, amino acid
PM03B	B03	L-Histidine	N-Source, amino acid
PM03B	B08	L-Phenylalanine	N-Source, amino acid
PM03B	B12	L-Tryptophan	N-Source, amino acid
PM03B	C03	D-Alanine	N-Source, amino acid

**Table 2:** PM conditions where the BM16 respired more than WT.

Plate	Well	Item tested	Mode of Action
PM01	B03	Glycerol	C-Source, carbohydrate
PM01	B10	Formic Acid	C-Source, carboxylic acid
PM01	D07	a-Ketobutyric Acid	C-Source, carboxylic acid
PM01	H12	Ethanolamine	C-Source, alcohol

**Table 3:** KEGG pathways in the liver that were significantly regulated (*p* < 0.05) by *B. pseudomallei* WT and BM16 infection.

Condition	KEGG pathway	*p*-value	
		**Liver-WT**	**Liver-BM**
Up-regulated	Phagosome	4.24E-17	2.60E-14
	Apoptosis	2.47E-08	1.03E-05
	Chemokine signalling pathway	3.49E-08	2.49E-04
	Cytokine-cytokine receptor interaction	5.27E-08	2.58E-07
	NOD-like receptor signalling pathway	1.77E-07	3.77E-06
	Natural killer cell mediated cytotoxicity	6.05E-06	2.92E-05
	Toll-like receptor signalling pathway	8.34E-06	2.92E-05
	B cell receptor signalling pathway	1.86E-03	3.69E-02
	Endocytosis	2.43E-03	1.77E-03
	RIG-I-like receptor signalling pathway	3.24E-03	3.68E-03
	Jak-STAT signalling pathway	1.10E-02	1.89E-02
	Regulation of actin cytoskeleton	2.85E-03	—a
	MAPK signalling pathway	4.17E-03	—
	P53 signalling pathway	5.62E-03	—
	Bacteria invasion of epithelial cells	1.61E-02	—
	Fc gamma R-mediated phagocytosis	—	3.75E-02
Down-regulated	PPAR signalling pathway	2.83E-11	4.76E-07
	Nitrogen metabolism	2.32E-05	1.03E-04
	ABC transporters	2.98E-03	6.68E-04
	Endocytosis	3.39E-03	—
	MAPK signalling pathway	1.00E-02	—
	Regulation of actin cytoskeleton	4.45E-02	—

^a^“—”: No significant pathway.

**Table 4:** KEGG pathways in the spleen that were significantly regulated (*p* < 0.05) by *B. pseudomallei* WT and BM16 infection.

Condition	KEGG pathway	*p*-value	
		Spleen-WT	Spleen-BM
Up-regulated	Cytokine-cytokine receptor interaction	1.74E-13	6.61E-16
	NOD-like receptor signalling pathway	4.33E-08	2.98E-07
	Chemokine signalling pathway	1.85E-06	4.57E-08
	Toll-like receptor signalling pathway	1.23E-04	1.20E-05
	Jak-STAT signalling pathway	9.35E-04	7.36E-05
	RIG-I-like receptor signalling pathway	1.33E-03	2.82E-04
Down-regulated	Cytokine-cytokine receptor interaction	2.89E-02	—a
	Phagosome	2.93E-02	—
	Chemokine signalling pathway	4.04E-02	—
	Lysosome	—	2.80E-02
	B cell receptor signalling pathway	—	4.11E-02
	Primary immunodeficiency	—	4.37E-02

^a^“—”: No significant pathway.
