# Supplementary material for: Eukaryotic pathways targeted by the type III secretion system effector protein, BipC, involved in the intracellular lifecycle of Burkholderia pseudomallei
Source: Sci Rep. 2016 Sep 16;6:33528. doi: 10.1038/srep33528 (PMC5025855; doi:10.1038/srep33528)
Supplement: Supplementary Information [file srep33528-s1.pdf]

Supplementary information

Eukaryotic pathways targeted by the type III secretion system effector protein, BipC, involved in the intracellular lifecycle of *Burkholderia pseudomallei*

Wen-Tyng Kang, Kumutha Malar Vellasamy and Jamuna Vadivelu*

Department of Medical Microbiology, Faculty of Medicine, University of Malaya, 50603, Kuala Lumpur, Malaysia.

* Corresponding author: jamuna@ummc.edu.my

Supplementary Figure S1. Heat map of phenotypic microarray (PM) for the comparison between *B. pseudomallei* wild type and *bipC* mutant infection.

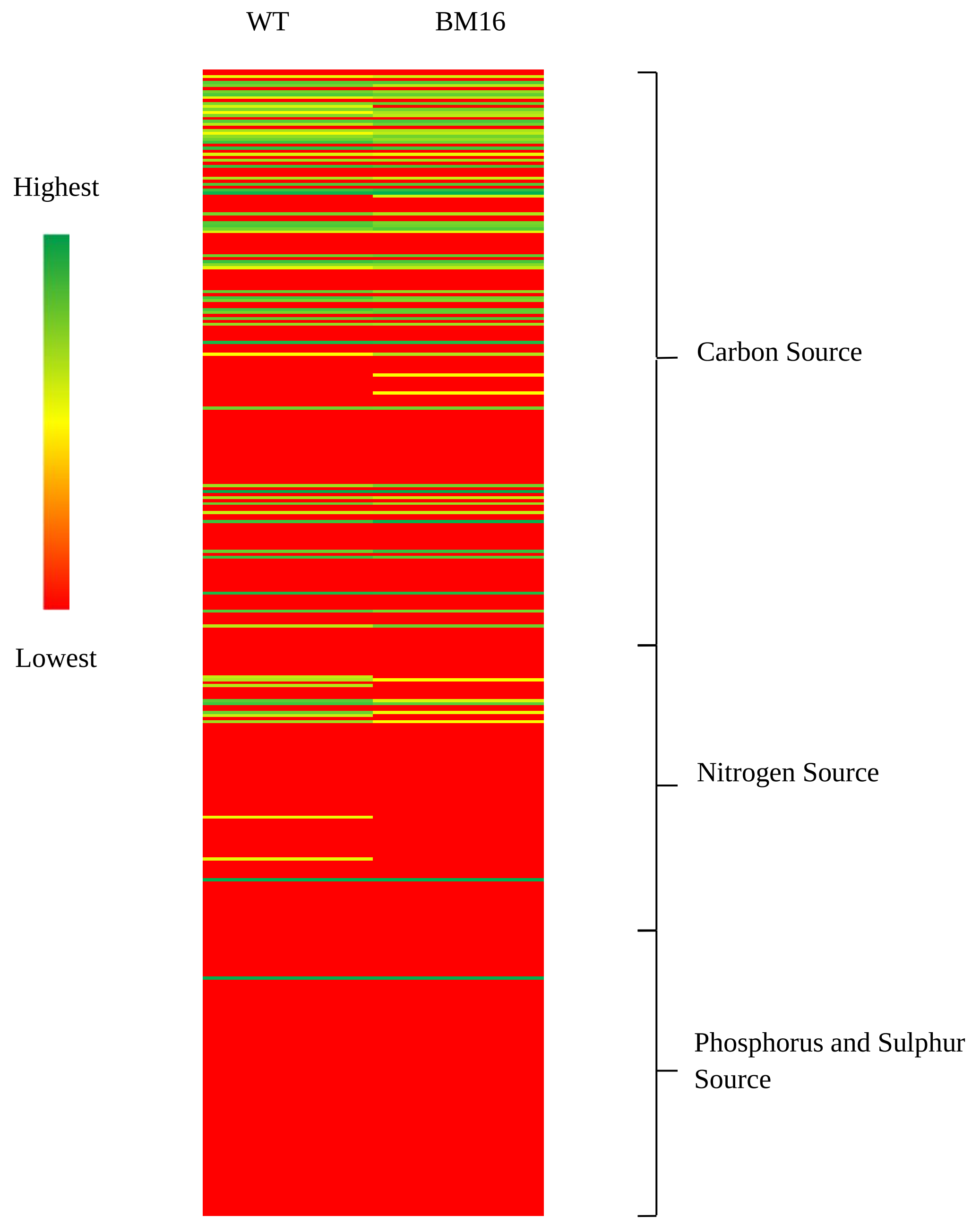

Phenotypic differences based on the ability of the WT and BM16 to utilise carbon, nitrogen, phosphorous, and sulphur sources. The area under the curve (AUC) for a selection of different substrates following 48 hours incubation in each condition is plotted here.

Supplementary Table S1: Kinetic growth curve for phenotypic microarray. Value of the average area under the kinetic growth curve for the substrates utilised by *B. pseudomallei* WT and BM16 in PM 1 to 4.

Sources	WT	BM16
**Negative Control PM1**	0	0
**N-Acetyl-D-Glucosamine**	1641	4143
**Succinic Acid**	15317	15862
**D-Galactose**	12590	6378
**L-Proline**	13142	9952
**D-Alanine**	16075	14321
**D-Trehalose**	2126	5803
**Dulcitol**	12167	14056
**D-Serine**	3515	0
**D-Sorbitol**	12082	13491
**Glycerol**	250	7801
**L-Fucose**	11529	6249
**D-Gluconic Acid**	15378	16679
**D,L-a-Glycerol-Phosphate**	6694	13142
**L-Lactic Acid**	8593	7917
**Formic Acid**	1046	5916
**D-Mannitol**	10201	13171
**L-Glutamic Acid**	13291	9611
**D-Glucose-6-Phosphate**	18049	13162
**D,L-Malic Acid**	19109	17178
**Tween 20**	3476	1887
**D-Fructose**	8979	8720
**a-D-Glucose**	18597	17169
**L-Asparagine**	7594	4588
**D-Glucosaminic Acid**	16294	16215
**Tween 40**	17845	19302
**a-Keto-Glutaric Acid**	22498	23377
**a-Keto-Butyric Acid**	0	4219
**L-Glutamine**	12701	7579
**D-Fructose-6-Phosphate**	15712	12390
**Tween 80**	16543	13404
**a-Hydroxy Glutaric Acid-g-Lactone**	12898	15991
**a-Hydroxy-Butyric Acid**	3396	2722
**m-Inositol**	13362	14791
**Fumaric Acid**	18233	16949
**Bromo-Succinic Acid**	10949	11198
**Propionic Acid**	2127	5749
**L-Serine**	14272	10134
**L-Alanine**	17322	12941
**L-Alanyl-Glycine**	12321	11535
**Mono Methyl Succinate**	17020	14632

**Methyl Pyruvate**	12656	14442
**L-Malic Acid**	14606	16139
**p-Hydroxy-Phenylacetic Acid**	9925	9150
**Pyruvic Acid**	21100	20714
**Ethanolamine**	209	7505
**Negative Control PM2**	0	0
**Gelatin**	0	512
**N-Acetyl-D-Galactosamine**	0	145
**D-Arabitol**	10408	12410
**Xylitol**	7144	12538
**g-Amino-Butyric Acid**	18415	22367
**Butyric Acid**	6695	5025
**Caproic Acid**	10815	8579
**Dihydroxy-Fumaric Acid**	3834	5078
**b-Hydroxy-Butyric Acid**	14166	21911
**Quinic Acid**	11090	17782
**Sebacic Acid**	14220	14418
**4-Hydroxy-L-Proline (trans)**	16379	19547
**L-Phenylalanine**	12290	11670
**D,L-Octopamine**	5034	12838
**Negative Control PM3**	0	0
**L-Glutamic AcidI**	4093	0
**L-GlutamineI**	5461	91
**L-HistidineI**	5150	0
**L-PhenylalanineI**	11423	2121
**L-ProlineI**	13082	9195
**L-Tryptophan**	10172	1017
**L-Tyrosine**	3646	0
**D-AlanineI**	6630	261
**N-Acetyl-D-GlucosamineI**	1300	0
**Xanthine**	1667	0
**g-Amino-N-Butyric Acid**	17361	14368
**Negative Control PM4**	0	0
**L-a -Phosphatidyl-D,L-Glycerol**	6427	3962

Supplementary Figure S2. LD_{50} for *B. pseudomallei* K96243 (WT).

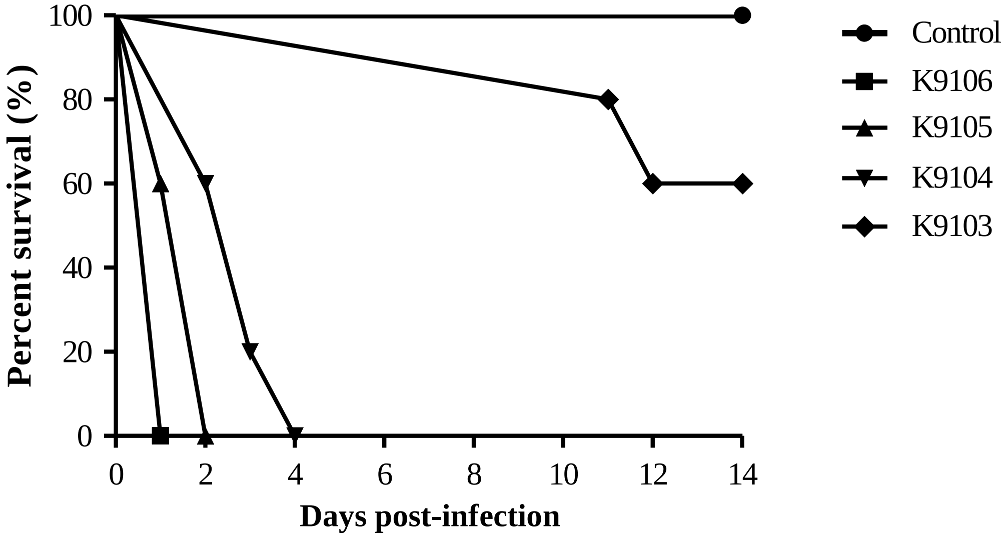

Mice were infected through i.p. route with different doses from 10^3 to10^6 CFU and observed daily up to 14 days. The percentage survival was plotted against time.

Supplementary Table S2: Heat map genes in the liver and spleen following infection with *B. pseudomallei* WT and BM16.

Gene Symbol	Liver-WT	Liver-BM16	Spleen-WT	Spleen-BM16
**Tnf**	55.98	25.67	-[a]	-
**Csf1**	4.72	4.08	-	-
**Fas**	4.19	2.98	-	-
**Il1b**	11.81	6.80	2.93	6.04
**Ccl2**	1191.08	137.88	135.93	93.41
**Il12rb1**	8.34	4.37	9.19	6.04
**Tnfrsf1a**	2.30	-	-	-
**Ifng**	27.48	25.05	100.39	177.29
**Il6**	47.56	15.82	171.84	236.29
**Csf2rb**	11.58	5.30	3.05	3.10
**Il1r2**	54.82	17.38	63.91	15.18
**Tnfrsf10b**	14.30	4.84	-	-
**Cxcl14**	19.13	23.08	-	-
**Ccr5**	3.50	2.84	-	-
**Ccl9**	4.44	-	-	-
**Ccl3**	41.35	5.65	7.74	5.83
**Ifngr2**	4.36	-	-	-
**Ifngr1**	4.79	-	-	-
**Cxcl16**	7.71	-	-	-
**Tnfrsf12a**	51.33	7.48	-	-
**Ccl19**	5.33	9.26	-	-
**Cxcl10**	584.34	186.23	20.16	25.48
**Osmr**	15.53	9.59	-	-
**Cxcr2**	19.00	8.93	4.49	3.10
**Il2rg**	12.15	10.60	-	-
**Cxcl9**	460.27	138.70	33.02	19.60
**Pdgfb**	3.72	-	-	-
**Ccl7**	394.74	129.16	578.00	372.36
**Cxcl1**	23.42	43.95	71.18	75.63
**Ccr8**	36.06	16.86	-	-
**Il6st**	2.63	2.82	-	-
**Ccl12**	25.45	15.65	105.82	65.34
**Ccl4**	44.46	-	6.14	5.65
**Ltb**	100.34	-	-	-
**Crlf2**	4.08	2.25	-	-
**Tnfsf10**	7.15	9.12	3.54	4.08
**Il1a**	-	-	8.75	11.10
**Ccl8**	-	-	13.53	8.22
**Il10**	-	-	18.57	3.29
**Csf3**	-	-	52.96	18.42
**Ccl11**	-	-	16.34	6.97
**Cxcl5**	-	-	18.22	23.93
**Ccl24**	-	-	3.88	4.01
**Ccl17**	-	-	11.51	3.68
**Cxcl2**	-	-	35.83	27.99
**Inhbb**	-	-	14.82	-
**Ifna12**	-	-	7.88	6.99
**Ccr6**	-	-	-5.09	-3.83
**Csf1r**	-	-	-2.43	-
**Ccr3**	-8.09	-8.48	-4.89	-3.41
**Cx3cr1**	-14.83	-17.26	-22.23	-6.05

**Tnfrsf13c**	-	-	-2.63	-2.69
**Tnfrsf13b**	-	-	-3.32	-2.62
**Stat3**	2.50	3.35	-	-
**Socs3**	10.27	11.88	7.39	5.10
**Pik3cd**	2.43	2.16	-	-
**Jak2**	3.52	-	-	-
**Bcl2l1**	4.77	-	-	-
**Csf2rb2**	3.16	3.02	-	-
**Socs1**	188.68	164.65	15.73	14.05
**Csf2**	-	-	9.36	28.14
**Stat2**	-	-	4.57	3.62
**Rac2**	4.22	2.54	-	-
**Dusp3**	2.05	-	-	-
**Dusp2**	10.64	3.24	-	-
**Map3k14**	6.46	4.92	-	-
**Ikbkg**	6.27	-	-	-
**Ddit3**	11.88	3.21	-	-
**Gadd45g**	3.22	6.85	-	-
**Mapkapk3**	5.45	-	-	-
**Nras**	3.63	-	-	-
**Gng12**	5.73	-	3.26	2.27
**Traf2**	2.99	2.15	-	-
**Rhoc**	12.34	-	-	-
**Rac1**	2.11	-	-	-
**Prkx**	4.95	2.76	-	-
**Nfkb1**	7.64	-	-	-
**Dusp5**	7.83	-	-	-
**Daxx**	4.42	4.16	4.10	3.14
**Nfkb2**	21.23	10.24	-	-
**Cd14**	365.39	116.24	-	-
**Mapk7**	3.59	-	-	-
**Relb**	25.13	12.26	-	-
**Gadd45b**	12.26	-	3.93	3.32
**Mapk8ip1**	-3.22	-2.70	-	-
**Map2k6**	-8.94	-6.31	-	-
**Ppp3cb**	-2.05	-	-	-
**Ppm1b**	-3.36	-2.68	-	-
**Abcg5**	-8.71	-7.37	-	-
**Abcc6**	-11.11	-2.97	-	-
**Abcb11**	-9.69	-3.51	-	-
**Abcb4**	-7.04	-	-	-
**Abcg8**	-33.87	-7.39	-	-
**Abca8a**	-88.25	-20.40	-	-
**Abcb6**	-5.85	-3.44	-	-
**Abcd3**	-5.54	-4.29	-	-
**Abcc3**	-	-	-3.33	-
**Abca1**	-	2.48	-	-2.05
**Hck**	5.78	3.65	-	-
**Was**	2.73	-	-	-
**Fgr**	5.15	3.42	-	-
**Nfkbib**	4.45	3.52	-	-
**Gng2**	5.14	-	-	-
**Gnb1**	3.06	-	-	-
**Nfkbia**	6.44	4.09	-	-
**Gnai2**	2.25	-	-	-
**Gm1987**	-15.80	-	-4.03	-
**Prkcb**	-	-	-2.89	-2.79

**Hsp90b1**	2.92	2.90	-	-
**Tnfaip3**	74.07	26.85	3.40	-
**Mefv**	12.44	-	-	-
**Ripk2**	34.65	24.12	-	-
**Birc2**	6.47	-	-	-
**Casp1**	3.67	2.99	-	-
**Nlrp1b**	-	-	2.04	-
**Mapk13**	-	-	5.39	4.91
**Gzmb**	14.03	6.40	8.41	7.44
**Bid**	11.79	6.03	-	-
**H2-T23**	4.19	3.61	-	-
**H2-K1**	5.03	4.05	-	-
**Plcg2**	2.80	2.34	-	-
**Icam1**	44.28	28.82	-	-
**Fcgr4**	6.44	5.32	14.02	13.15
**H2-D1**	4.80	3.56	-	-
**Itgal**	2.43	3.00	-	-
**Tyrobp**	2.31	-	-	-
**Fcer1g**	2.76	2.06	-	-
**Myd88**	5.04	5.99	-	-
**Lbp**	3.89	7.86	-	-
**Tlr2**	60.85	54.59	-	-
**Irf7**	9.45	12.95	10.80	9.86
**Tlr8**	9.17	3.73	-	-
**Irf5**	8.16	4.23	-	-
**Jun**	-	-	3.29	2.34
**Ifitm1**	3.85	4.13	-	-
**Nfkbie**	25.19	7.36	-	-
**Pik3ap1**	3.38	4.29	-	-
**Dapp1**	10.45	-	-	-
**Cd81**	-	-	-	-2.02
**Cr2**	-	-	-4.32	-2.60
**Isg15**	26.01	31.49	9.45	10.27
**Dhx58**	7.56	9.60	5.52	4.85
**Tmem173**	6.21	5.48	-	-
**Ifih1**	-	-	4.49	4.10
**Pip5k1a**	28.03	26.65	23.04	7.27
**Cfl1**	2.37	2.03	0	-
**Gm5637**	6.36	3.24	0	-
**Fcgr1**	4.36	3.65	4.33	4.05
**Asap3**	3.43	5.63	-	-
**Apoa5**	-44.35	-	-	-
**Acsl1**	-7.24	-8.14	-	-
**Ehhadh**	-9.14	-9.68	-	-
**Cpt1a**	-3.49	-	-	-
**Apoc3**	-6.24	-	-	-
**Slc27a2**	-17.38	-3.98	-	-
**Scp2**	-4.36	-	-	-
**Slc27a5**	-4.70	-	-	-
**Ppara**	-3.54	-2.89	-	-
**Dbi**	-2.88	-	-	-
**Acadm**	-3.72	-2.28	-	-
**Cyp4a10**	-82.15	-13.64	-	-
**Cyp4a14**	-213.07	-39.90	-	-
**Acaa1b**	-9.21	-3.68	-	-
**Fads2**	-4.64	-2.89	-	-
**Cyp8b1**	-238.60	-	-	-

**Hmgcs2**	-17.44	-3.89	-	-
**Cyp7a1**	-8.17	-222.65	-	-
**Apoa2**	-2.71	-	-	-
**Fabp1**	-10.79	-3.72	-	-
**Cyp4a32**	-67.73	-12.41	-	-
**Acox1**	-5.57	-	-	-
**Cyp4a31**	-73.85	-12.46	-	-
**Atp6v0a4**	50.42	53.91	-	-
**Atp6v0e**	2.71	-	-	-
**Tap1**	14.57	17.72	-	-
**C1ra**	2.54	2.12	2.60	-
**H2-M3**	5.84	3.43	-	-
**Tuba1b**	2.69	-	-	-
**Tuba1c**	3.18	-	-	-
**H2-Eb1**	5.83	4.44	-	-
**Tubb6**	19.33	-	-	-
**M6pr**	2.39	-	-	-
**H2-T10**	3.35	2.81	-	-
**H2-Q8**	4.88	4.13	-	-
**Olr1**	15.20	-	-	-
**Itga2**	31.67	-	-	-
**Tubb5**	6.73	3.00	-	-
**Tubb3**	16.18	12.57	16.88	25.27
**Msr1**	3.04	2.07	-	-
**Ncf4**	4.74	2.99	-	-
**Tubb2b**	49.91	-	-	-
**H2-T9**	3.53	2.76	-	-
**H2-Q2**	4.34	3.67	-	-
**Cyba**	6.39	3.46	-	-
**Tuba8**	48.97	13.77	-	-
**Arf6**	2.77	-	-	-
**Arf3**	2.07	-	-	-
**Rab35**	2.06	-	-	-
**Rab7**	2.15	-	-	-
**Sec22b**	2.79	2.57	-	-
**Marco**	41.29	12.31	-	-
**Cybb**	12.46	4.82	-	-
**H2-Aa**	4.30	4.32	-	-
**H2-T24**	3.20	5.82	3.09	3.79
**H2-DMb1**	7.32	7.47	-	-
**Sec61b**	2.06	2.42	-	-
**LOC547349**	4.90	4.04	-	-
**Sec61a1**	2.53	2.81	-	-
**H2-Ab1**	5.82	5.76	-	-
**Tap2**	10.59	8.40	2.49	-
**Sec61g**	2.66	3.36	-	-
**Stx18**	-	2.29	-	-
**Actb**	2.65	-	-	-
**Tubal3**	-	-	-2.15	-2.41
**Cd209d**	-	-	-4.19	-2.61
**Colec11**	-	-	-4.73	-
**Itgb5**	-	-	-3.42	-3.43
**Mrc1**	-	-	-2.84	-3.85
**Arf6**	2.77	-	-	-
**Ehd1**	6.88	-	-	-
**Arfgap3**	4.19	4.11	-	-
**Ehd4**	3.20	2.53	-	-

**Arfgap1**	2.54	2.80	-	-
**Pml**	2.01	2.90	-	2.21
**Adrb2**	24.67	6.86	-	-
**Mdm2**	6.07	-	-	-
**Pdcd6ip**	2.0	-	-	-
**Ehd3**	-3.78	-3.87	-	-
**Sh3glb2**	-2.65	-	-	-
**Acp5**	-	-	-	-2.18
**Hexb**	-	-	-	-2.21
**Sort1**	-	-	-2.24	-2.48
**Sgsh**	-	-	-2.20	-3.33
**Irak3**	48.09	26.43	-	-
**Cflar**	3.92	-	3.84	-
**Bax**	4.72	-	0	-
**Cd82**	2.41	-	0	-
**Cdk4**	2.59	-	0	-
**Ccne1**	5.71	3.15	0	-
**Serpine1**	816.03	202.01	25.49	16.41
**Shisa5**	4.33	3.88	-	-
**Msn**	2.89	2.01	-	-
**Insrr**	41.90	35.61	-	-
**Iqgap1**	3.78	-	-	-
**Myh9**	2.70	-	-	-
**Gna13**	25.34	-	-	-
**Wasf2**	2.75	-	-	-
**Arpc1b**	5.57	-	-	-
**Ezr**	2.06	3.03	-	-
**Ssh1**	3.80	2.53	-	-
**Arpc5**	2.29	-	-	-
**Mylk**	-4.07	-	-	-
**Araf**	-3.30	-	-	-
**Myl9**	-13.38	-4.86	-5.13	-2.12
**Itga8**	-26.46	-11.02	-	-
**Hcls1**	4.09	3.00	-	-
**Cttn**	2.22	-	-	-
**Arpc1b**	5.57	-	-	-
**Arpc5**	2.29	-	-	-
**Cth**	-6.53	-4.30	-	-
**Hal**	-16.55	-2.78	-	-
**Car1**	-7.56	-12.30	-	-
**Car5a**	-276.51	-	-	-
**Glud1**	-5.64	-2.94	-	-
**Car3**	-1912.19	-82.55	-	-
**Gls2**	-9.18	-3.87	-	-
**Car2**	-5.07	-	-	-

[a] ‘-‘, no significant change in gene.

Supplementary Table S3. Primer sequences of the genes used for microarray validation.

**Primers**	**Gene symbol description**	**Primer Sequence (5' – 3')**	**Amplicon size (bp)**
GAPDH (F)	Glyceraldehyde 3-phosphate dehydrogenase	AGG TCG GTG TGA ACG GAT TTG	95
GAPDH (R)		GGG GTC GTT GAT GGC AAC A	
Fas (F)	TNF receptor superfamily member 6	GCG GGT TCG TGA AAC TGA TAA	61
Fas (R)		GCA AAA TGG GCC TCC TTG ATA	
Rac1 (F)	RAS-related C3 botulinum substrate 1	GAG ACG GAG CTG TTG GTA AAA	138
Rac1 (R)		ATA GGC CCA GAT TCA CTG GTT	
IL16 (F)	Interleukin 16	AAG AGC CGG AAA TCC ACG AAA	116
IL16 (R)		GTC TCA AAA GGG TCA GGG TAC T	
Cflar (F)	CASP8 and FADD-like apoptosis regulator	TGG CTG AAT TGC TCT ACA GAG T	105
Cflar (R)		CCA GGT GAG GGT TTC TGC G	
Cyp2e1 (F)	Cytochrome P450, family 2, subfamily e, polypeptide 1	CGT TGC CTT GCT TGT CTG GA	105
Cyp2e1 (R)		AAG AAA GGA ATT GGG AAA GGT CC	
Nlrp6 (F)	NLR family, pyrin domain containing 6	CTC GCT TGC TAG TGA CTA CAC	193
Nlrp6 (R)		AGT GCA AAC AGC GTC TCG TT	
Inhbb (F)	Inhibin beta-B	GAC GCC TTA ACC TGG ATG TG	140
Inhbb (R)		GCG GAT GCG ATG TCT GCT AT	
Tnf (F)	Tumor necrosis factor	CCT GTA GCC CAC GTC GTA G	148
Tnf (R)		GGG AGT AGA CAA GGT ACA ACC C	

Abbreviations: F - forward primer; R - reserve primer
